# The Happy Teen programme: a holistic outpatient clinic‐based approach to prepare HIV‐infected youth for the transition from paediatric to adult medical care services in Thailand

**DOI:** 10.7448/IAS.20.4.21500

**Published:** 2017-05-16

**Authors:** Rangsima Lolekha, Vitharon Boon‐yasidhi, Yossawadee Na‐Nakorn, Boonying Manaboriboon, Warunee Punpanich Vandepitte, Michael Martin, Jariya Tarugsa, Wipada Nuchanard, Pimsiri Leowsrisook, Ketwadee Lapphra, Piyarat Suntarattiwong, Vorapathu Thaineua, Kulkanya Chokephaibulkit, Happy Teen Working Group

**Affiliations:** ^1^ Division of Global HIV and Tuberculosis Thailand/Asia Regional Office, Thailand Ministry of Public Health (MOPH) – US Centers for Disease Control and Prevention (CDC) Collaboration, Nonthaburi, Thailand; ^2^ Department of Pediatrics, Siriraj Hospital, Mahidol University, Bangkok, Thailand; ^3^ Queen Sirikit National Institute of Child Health, Thailand MOPH, Bangkok, Thailand

**Keywords:** HIV, adolescent, treatment, knowledge, transition, adherence, Thailand

## Abstract

**Introduction**: We developed an 18‐month Happy Teen 2 (HT2) programme comprised of a one‐day workshop, two half‐day sessions, and three individual sessions to prepare HIV‐infected youth for the transition from paediatric to adult HIV care services. We describe the programme and evaluate the change in youth's knowledge scores.

**Methods**: We implemented the HT2 programme among HIV‐infected Thai youth aged 14–22 years who were aware of their HIV status and receiving care at two hospitals in Bangkok (Siriraj Hospital, Queen Sirikit National Institute of Child Health [QSNICH]). Staff interviewed youth using a standardized questionnaire to assess HIV and health‐related knowledge at baseline and at 12 and 18 months while they participated in the programme. We examined factors associated with a composite knowledge score ≥95% at month 18 using logistic regression.

**Results**: During March 2014–July 2016, 192 of 245 (78%) eligible youth were interviewed at baseline. Of these, 161 (84%) returned for interviews at 12 and 18 months. Among the 161 youth, the median age was 17 years, 74 (46%) were female, and 99% were receiving antiretroviral treatment. The median composite score was 45% at baseline and increased to 82% at 12 months and 95% at 18 months (*P* < 0.001). The range of median knowledge scores for antiretroviral management, HIV monitoring, HIV services, and family planning significantly increased from baseline (range 0–75%) to (range 67–100%) at 12 months and to 100% at 18 months (*P* < 0.001). Almost all youth were able to describe education and career goals at 12 and 18 months compared to 75% at baseline. In multivariable analysis, a composite knowledge score at 18 months >95% was associated with education level >high school (aOR: 2.15, 95%CI, 1.03–4.48) and receipt care at QSNICH (aOR: 2.43, 95%CI, 1.18–4.98). Youth whose mother and father had died were less likely to have score ≥95% (aOR: 0.22, 95%CI, 0.07–0.67) than those with living parents.

**Conclusions**: Knowledge useful for a successful transition from paediatric to adult HIV care increased among youth participating in the HT2 programme. Youth follow‐up will continue to assess the impact of improved knowledge on outcomes following the transition to adult care services.

## Introduction

In Thailand in 2015, more than 75% of perinatally HIV‐infected children were 10 years old or older. This public health achievement, based on universal access to medical care and antiretroviral therapy (ART), has given rise to a number of challenges [1–4]. Challenges include how to support these youth as they transition from paediatric HIV services to adult HIV care settings, ensuring they keep appointments, continue to adhere to life‐saving medications, and maintain their health, as they assume a greater, more independent role in their healthcare. Research and manuals describing interventions to promote self‐care and ART adherence, increase youth self‐esteem, promote family planning, and reduce sexual risk behaviours among youth transitioning from paediatric to adult HIV care services have been published, but mainly from resource‐rich settings [1,5–8].

During 2009–2011, the Happy Teen Working Group, a multidisciplinary team including specialists in psychology, adolescent medicine, HIV medicine, and counsellors from Siriraj Hospital, Queen Sirikit National Institute of Child Health (QSNICH), and the Thailand MOPH‐U.S. CDC Collaboration (TUC) developed an intervention programme for HIV‐positive youth called the Happy Teen 1 (HT1) programme [9]. The programme was developed for paediatric practices that were caring predominantly for perinatally HIV‐infected children and youth. Children aged 12 years old or older were offered two groups (game‐based activities) and two individual sessions during four consecutive routine clinic visits for HIV care (usually every 2–3 months). The sessions focused on four main areas including health knowledge, coping skills, sexual risk reduction, and life goals. The aim was to address these issues in HIV‐infected youth in an effort to reduce behavioural risk and secondary HIV transmission [9]. An evaluation of the HT1 programme in 2011[10], showed that participation was associated with a modest but statistically significant improvement in knowledge and attitudes regarding ART management, reproductive health, and HIV‐associated risk behaviours, but the intervention did not have an effect on alcohol use or sexual behaviour and did not address issues related to the transition from youth to adult HIV care services. The study results suggested that more comprehensive interventions were needed to promote safe behaviours in these youth and to prepare them for transition to adult medical services [10].

During 2012–2014, the Happy Teen Working Group developed a hospital outpatient clinic‐based comprehensive programme for perinatally HIV‐infected youth (i.e. The Happy Teen 2 (HT2) programme) that built on the experience with the HT1 programme. The HT2 programme aims to be a holistic model to help older adolescents, 14–21 years old, prepare for adulthood including the transition from paediatric to adult HIV care services and provides tools to improve communication skills with caretakers and explore and develop life goals. In this report, we describe the HT2 programme and evaluate changes in youth's knowledge after participating in the HT2 programme.

### Methods

During March 2014–July 2016, all perinatally HIV‐infected youth aged 14–22 years old who received care at paediatric HIV clinics of two tertiary care hospitals (QSNICH in central Bangkok and Siriraj Hospital located on the outskirts of Bangkok) and were aware of their HIV status [11] were invited to participate in the HT2 programme. HT2 participants were not required to have completed the HT1 programme. Youth caretakers and youth aged 18 years and older completed the informed consent process. Youth aged less than 18 years old provided informed assent. Youth and caretakers who did not wish to participate in the programme received routine individual counselling during clinic visits.

The HT2 programme aims to assess and improve skills and knowledge in four strategies areas consistent with the HT1 programme [9,10,12]: health knowledge, coping skills, sexual risk reduction, and life goals (Table 1) but the activities were designed for older youth. Interventions included a one‐day weekend workshop, two half‐day meetings, and three individual sessions. Details of interventions and the flow of activities are described in Tables 1 and 2. The one‐day weekend workshop (7.5 h) was an extra visit (i.e. in addition to routine medical visits) at the hospitals or offsite. Caretakers who participated in the one‐day workshop attended for a total of 5.5 h. Session activities included reviewing the importance and benefits of strong family relationships and communication, parenting, and the importance of preparing youth for the transition to adult medical care, higher education, and careers. The two half‐day meetings (2–3 h) and three individual sessions were organized during regular HIV care clinic visits (usually every 2–3 months) and were conducted by project staff. The individual sessions lasted 30–60 min [9] and allowed counsellors to explore participants individual risks, assess prerequisite knowledge for transition to adult HIV care, provide reproductive health and risk reduction counselling, promote increased self‐esteem, and provide psychological support to HIV‐positive youth. If a youth was unable to answer a question correctly, project staff would provide the correct answers, discuss techniques to remember information, and how to find information needed for a successful transition to an adult medical clinic. Youth with risk behaviours identified during the individual sessions were referred to specialists based on their needs.

**Table 1:** Four strategies of the Happy Teen Programs.

	Strategy No. Age range	Happy Teen 1 Program 12‐21 years old	Happy Teen 2 Program 14‐22 years old
1	“Health Knowledge” (Knowledge about HIV‐positive youth health)	Pubertal changes and hygiene Sex education (e.g., reproductive health, prevention of unintended pregnancy and sexually transmitted diseases, and condom use) ART adherence	Transitioning to adult HIV care Self‐care (e.g., ART adherence, keeping appointments) Knowledge about Health Care Insurance
2	“Coping Skills” (Self‐esteem and stress management promotion)	Communication skills (e.g., negotiation, how to say no to peers) Problem solving skills and stress management	Communication skills Self‐esteem Effective parenting for youth
3	“Sexual Risk Reduction” (Sex education and risk reduction counseling)	Dating, boyfriend/girlfriend relationships Abstinence/delay sexual relationships until appropriate age	Safe sex behaviors Relationships Reproductive health, family planning, and healthy conception Reduce risk behaviors for HIV and STIs HIV‐disclosure and partner testing
4	“Life Goals” (Life skills and responsibilities promotion)	Appropriate use of computer, internet, chat lines Honest behavior, following rules Making friends, schooling Avoiding substance abuse, gambling, and other risky behavior	Education planning Career planning

**Table 2:** Happy Teen 2 Program activities, target audience, and contents.

			Target audiences	
Session	Topics	Content	Youth	Care‐taker
1. One‐day weekend workshop	Session#1: Introduction to the program + ice breaker + video (1 hr)	‐ Rational for and principles of readiness preparation for transitioning youth to adulthood and adult HIV care service	√	√
	Session#2: Strengthening family & communication skills for youth and caretaker (1 hr 45 min)	‐ Review the importance and benefits of strong family relationships and communication ‐ Guidance on how to strengthen family communication ‐ Discuss how to develop good family relationships and effective communication	√	√
	Session#3: Self‐esteem (1 hr)	‐ Importance of self‐esteem ‐ Assess your self‐esteem and strengthen your self‐esteem	√	
	Session#4: Education and career (2 hr) youth: basic vocational orientation test (2 hrs) Caretaker: (1 hr); then parenting class	‐ Build positive attitudes and aspirations towards educational attainment and careers ‐ Provide resources for formal and informal education ‐ Provide information about career planning, qualifications of various professions, and requirements for HIV blood testing on job applications ‐ Assess personal aptitude and competency to do a certain kinds of work or careers	√ √	
	Session#5: Parenting of youth (2 hrs)	‐ Basic knowledge and skills to provide care to HIV positive youth ‐ How to provide sex education to youth ‐ How to develop plans to resolve youth problems		√
2. Half day meeting#1	Session#6 : Self‐care and ART management (advanced) including transition to adult care, health care benefit package (2 hrs)	‐ Review self‐history of HIV treatment ‐ How to come to clinic and make clinic appointments by yourself ‐ Abnormal symptoms that need follow‐up ‐ Self‐care and self‐management on ART ‐ Health care insurance ‐ Transition to adult HIV care services	√	
3. Half day meeting#2	Session# 7: Reproductive health & family planning + reduce risk behavior for HIV & STIs transmission + self‐disclosure & partner testing	‐ Sexually transmitted infections knowledge ‐ Safe sex practices ‐ Family planning, contraception ‐ Marriage planning, safe conception, planned pregnancy, Prevention of mother‐to‐child transmission of HIV ‐ Prevention of unplanned pregnancies ‐ HIV disclosure and partner HIV testing	√	
4. Individual session#1	Assess youth readiness for transition to adult HIV care services, youth problems, and provide counseling tailored to individual youth		√	
5. Individual session#2	Follow‐up youth readiness for transition, youth problems, and provide counseling tailored to individual youth		√	
6. Individual session#3	Follow‐up on youth problems, and provide counseling tailored to individual youth, transition ceremony with certificate		√	

The youth were interviewed by trained research staff using a standardized questionnaire (i.e. 22 questions) to assess knowledge of health‐related factors important for a successful transition to adult HIV care at baseline, 12, and 18 months (at the beginning of the second and third individual counselling sessions). The prerequisite knowledge for transition assessment form was modified from a transition tool developed by the New York State Department of Health [1] and Guidance for Transitioning Youth to Adult HIV Care Services developed by Thailand MOPH [13]. Following the assessment, research staff provided information about the transition to adult care covered in the questionnaire to the youth.

Staff used double data entry to extract data from questionnaires and entered the data in a Microsoft Access database. ART, ART adherence, CD4 count and viral load data (within 6 months) were abstracted from participants’ medical records and included in the database for analysis.

### Data analysis

Data were analyzed using SPSS 20.0 (Chicago, USA). Characteristics of perinatally HIV‐infected youth were reported as proportions (percentages). Cochran's Q Test was used to compared changes in proportion of youth who were able to answer prerequisite knowledge questions correctly at baseline, 12, and 18 months. Knowledge scores were calculated from the number of questions that a youth answered correctly (a full score was 22) as the percentage of correct answers and the Friedman Test was used to compare scores at baseline, 12, and 18 months. The Bonferroni correction used to account for multiple testing (i.e. 0.05/22); the significance cut point (α = 0.05) was *P* <0.0028. The mean percentages of knowledge scores were calculated and categorized by knowledge area. The consensus opinion of the Happy Teen working group, was that a composite score >95% would support a successful transition from paediatric to adult care services. Factors associated with a knowledge score >95% at month 18 were analyzed using logistic regression to estimate odds ratios (OR) with 95% confidence intervals (CI). Variables in bivariable analysis with a *P*‐value <0.10 were included in a multivariable analysis.

### Human subjects protections

Ethical Review Committees of QSNICH, Siriraj Hospital, the Thailand Ministry of Public Health (MOPH), and the U.S. Centers for Disease Control and Prevention Institutional Review Board reviewed and approved the study protocol and trial materials.

## Results

During March 2014–July 2016, 245 of 262 (94%) youth at the study sites met eligibility criteria and were invited to participate in the programme. Of these, 192 (78%) completed the consent process and were interviewed at baseline. The most common reasons given for not participating were that the individual did not have time, was unwilling, or was planning to transfer to adult care soon. Of the 192 youth who consented, 93 (48%) participated in the one‐day workshop; caretakers were invited to join youth at the one‐day workshop and 56 (29%) caretakers attended. A total of 124 (65%) youth attended the first half‐day meeting, 125 (65%) the second half‐day meeting, 174 (91%) the first individual session, 175 (91%) the second individual session and interview at 12 months, and 165 (86%) the third individual session and interview at 18 months; 56 (29%) youth attended all sessions and completed the entire programme. Of the 192 youth enrolled, 161 (84%) completed the interviews at baseline, 12, and 18 months and were included in the analyses.

Overall, youth reported high satisfaction with the sessions: 58 (94%) of 62 youth reported high satisfaction with the one‐day workshop, 94 (85%) of 111 with the first half‐day meeting, 108 (98%) of 110 with the second half‐day meeting, and 139 (98%) of 142 with the individual session. Participants from QSNICH were more likely to complete the entire programme than participants from Siriraj Hospital (41 [46%] at QSNICH compared with 15 [21%] at Siriraj Hospital; *P* <0.01). Among the 31 youth who did not participate in the interview at 12 or 18 months, 17 (55%) had been referred to an adult clinic, 8 (26%) were lost to follow‐up, 3 (10%) were not available, 2 (6%) had died, and 1 (3%) was in prison. Characteristics of 161 perinatally HIV‐infected youth are shown in Table 3. Median age was 17 years (range: 14–22) and 74 (46%) were female. Participants had been aware of their HIV status for a median of 56 months. Most youth (160 [99%]) had received ARV treatment and 125 (78%) had plasma HIV RNA concentrations <40 copies/ml. Median baseline CD4 lymphocyte count was 554 cells/mm^3^. Compared to QSNICH, participants enrolled at Siriraj Hospital had a higher baseline CD4 count, were aware of their HIV status for longer period of time, and had a higher literacy level (Table 3).

**Table 3:** Characteristics of perinatally HIV‐infected youth participating in the Happy Teen 2 Program by site and sessions completed, Thailand 2015‐2016.

	Number (%) or median (IQR)						
		Sites			Session participation		
Characteristics	Total (*N* = 161)	QSNICH^a^ (*N* = 89, 55.3%)	Siriraj (*N* = 72, 44.7%)	*P* **‐value**	All sessions (*N* = 56, 34.8%)	Not all sessions (*N* = 105, 65.2%)	*P* **‐value**
**1. Age: median (range) (years)**	17 (14, 22)	17 (14, 22)	17.5 (14, 22)	0.053	16 (15, 19)	17 (16, 19)	0.140
**2. Site**							
Siriraj Hospital	‐	‐	‐	‐	15 (26.8)	57 (54.3)	
QSNICH	‐	‐	‐	‐	41 (73.2)	48 (45.7)	0.001
**3. Gender: n (%)**							
Female	74 (46.0)	37 (41.6)	37 (51.4)		26 (46.4)	48 (45.7)	
Male	87 (54.0)	52 (58.4)	35 (48.6)	0.214	30 (53.6)	57 (54.3)	0.931
**4. Occupation: n (%)**							
Student, non‐formal education student	133 (82.6)	75 (84.3)	58 (80.6)		47 (83.9)	86 (81.9)	
Employee	14 (8.7)	6 (6.7)	8 (11.1)		4 (7.1)	10 (9.5)	
Unemployed	14 (8.7)	8 (9)	6 (8.3)	0.619	5 (8.9)	9 (8.6)	0.877
**5. Highest education attained: n (%)**							
Primary school or junior high school	74 (50.0)	40 (44.9)	34 (47.2)		29 (51.8)	45 (42.9)	
High school or vocational school	68 (42.2)	39 (43.8)	29 (40.3)		24 (42.9)	44 (41.9)	
Bachelor degree or higher vocational school	19 (11.8)	10 (11.2)	9 (12.5)	0.945	3 (5.4)	16 (15.2)	0.160
**6. Literacy level: n (%)**							
Very good	137 (85.1)	68 (76.4)	69 (95.8)		48 (85.7)	89 (84.8)	
Fair reading and writing	21 (13.0)	18 (20.2)	3 (4.2)		7 (12.5)	14 (13.3)	
Illiterate	3 (1.9)	3 (3.4)	0 (0)	0.002	1 (1.8)	2 (1.9)	0.987
**7. Main caretaker(s): n (%)**							
Father and/or mother	73 (45.3)	34 (38.2)	39 (54.2)		18 (32.1)	55 (52.4)	
Other relatives	74 (46.0)	45 (50.6)	29 (40.3)		33 (58.9)	41 (39)	
Other (e.g., non‐relatives, orphanage, self‐care)	14 (8.7)	10 (11.2)	4 (5.6)	0.066	5 (8.9)	9 (8.6)	0.040
**8. Parents Status: n (%)**							
Both alive	38 (23.8)	17 (19.3)	21 (29.2)		6 (10.7)	32 (30.8)	
Both dead	38 (23.8)	23 (26.1)	15 (20.8)		16 (28.6)	22 (21.2)	
One of the parents dead, unknown	84 (52.5)	48 (54.5)	36 (50)	0.326	34 (60.7)	50 (48.1)	0.017
**9. Household income (per month): n (%)**							
Less than or equal to 20,000 baht	124 (77)	74 (83.1)	50 (69.4)		48 (85.7)	76 (72.4)	
More than 20,000 baht	37 (23)	15 (16.9)	22 (30.6)	0.040	8 (14.3)	29 (27.6)	0.055
**10. Mode of HIV transmission: n (%)**							
Mother‐to‐child	158 (98.1)	89 (100)	69 (95.8)		56 (100)	102 (97.1)	
Blood transfusion, needle sharing, unknown	3 (1.9)	0 (0)	3 (4.2)	0.052	0 (0)	3 (2.9)	0.202
**11. Time from disclosure of HIV status to enrollment (months)^1^: Median (IQR)**	56 (34.5, 85)	41 (29, 82.5)	61.5 (44, 87)	0.006	54 (34.3, 89)	58 (34.5)	0.482
**12. Time in HIV care (years): Median (IQR)**	13 (9, 15.5)	13 (8, 15)	13.5 (10,16)	0.088	14 (10.3, 16)	13 (9,15)	0.142
**13. Time on ART (years): Median (IQR)**	13 (9, 15)	13 (9, 14.5)	13 (10, 15)	0.239	14 (10, 15)	12 (9, 15)	0.180
**14. Received ART: n (%)**	160 (99.4)	88 (98.9)	72 (100)	0.367	55 (98.2)	105 (100)	0.348
**15**. **HIV viral load level <40 copies/ml**	125 (77.6)	68 (76.4)	57 (79.2)	0.676	44 (78.6)	81 (77.1)	0.836
**16. CD4 count at baseline: Median (IQR)**	554 (367, 745)	488 (319, 660)	629 (444, 823)	0.002	540 (327, 672)	560 (391, 760)	0.170
**17. Participated in HT1 Program: n (%)**	53 (32.9)	27 (30.3)	26 (36.1)	0.438	20 (35.7)	33 (31.4)	0.582
**18. Participated in HT2 Program**							
**One‐day workshop**	79 (49.1)	45 (50.6)	34 (47.2)	0.673	56 (100)	23 (21.9)	<0.01
**First half‐day workshop**	111 (68.9)	72 (80.9)	39 (54.2)	<0.01	56 (100)	55 (52.4)	<0.01
**Second half‐day workshop**	111 (69.4)	81 (91)	30 (42.3)	<0.01	56 (100)	55 (52.9)	<0.01
**First individual session**	151 (93.8)	89 (100)	62 (86.1)	<0.01	56 (100)	95 (90.5)	0.015
**Second individual session**	161 (100)	89 (100)	72 (100)	N/A	56 (100)	105 (100)	N/A
**Third individual session**	161 (100)	89 (100)	72 (100)	N/A	56 (100)	105 (100)	N/A

***Note*** : *QSNICH: Queen Sirikit National Institute of Child Health, IQR: Interquartile range *^1^ Duration between disclosure and enrollment* *HT: Happy Teen*

Overall health‐related knowledge scores increased for all categories from baseline to month 18 (Table 4). The median score for prerequisite knowledge was 10 of 22 (45%) at baseline and increased to 18 of 22 (82%) at 12 months, and 21 of 22 (95%) at 18 months (*P* <0.001). At baseline, 90% of youth stated that they were able to manage and take their ART, 79% reported >95% ART adherence, 67% knew their health insurance scheme, 87% knew the processes involved in care services (e.g. registration, appointment, payments, medication retrieval), 78% knew at least two birth control methods, 80% knew how to prevent STI and HIV transmission, and 75% knew how to get information about education and careers. Only 14% of youth were able to correctly answer questions related to HIV viral load and CD4 count monitoring, 22% knew the date of their next medical appointment and their physician's name, and 27% knew how to correctly put on a condom at baseline. At baseline, males were more likely than females to correctly answer questions about their ART, ART side effects, the meaning of viral load, the name of their physician, next appointment date, the process of making medical appointments, and how to put on a condom (*P* <0.05)(data not shown).

**Table 4:** Assessment of health‐related knowledge of perinatally HIV‐infected youth participating in the Happy Teen 2 programme at baseline, 12, and 18 months, Thailand, 2015–2016.

Prerequisite knowledge for transition to adult HIV care	Baseline ***N* = 161** *n* (%)	12 months ***N* = 161** *n* (%)	18 months ***N* = 161** *n* (%)	*P* **‐value^a^**
Self‐care and Transition to Adult HIV Care	Number (%) of youth who answered correctly			
**ART management (full score 4)/Median score (IQR)**	3 (2,3)	3 (3,4)	4 (3,4)	<0.001*
**% median composite score (%IQR)**	75 (50, 75)	75 (75, 100)	100 (75, 100)	
State the names of your current ARVs	52 (32.3)	105 (65.2)	129 (80.1)	<0.001
Know how to manage ARV (taking, preparing, and storing)	145 (90.1)	159 (98.8)	161 (100)	<0.001
Have good (>95%) ARV adherence without assistance	127 (78.9)	156 (96.9)	159 (98.8)	<0.001
State the side effects and allergic symptoms of ARV, and if you have allergies to any medications	68 (42.2)	124 (77.0)	146 (90.7)	<0.001
**HIV monitoring (full score 6)/Median score (IQR)**	0 (0, 1)	4 (2,6)	6 (4,6)	<0.001*
**% median composite score (%IQR)**	0 (0, 16.7)	66.7 (33.3, 100)	100 (66.7, 100)	
State the meaning of CD4	58 (36.0)	134 (83.2)	152 (94.4)	<0.001
Know normal value of CD4 and how often it should be tested	22 (13.7)	112 (69.6)	140 (87.0)	<0.001
Know your CD4 count	20 (12.4)	104 (64.6)	134 (83.2)	<0.001
State the meaning of viral load (VL)	22 (13.7)	97 (60.2)	135 (83.9)	<0.001
Know value (for good viral load suppression) and how often it should be tested	13 (8.1)	79 (49.1)	126 (78.3)	<0.001
Know your VL level	11 (6.8)	76 (47.2)	120 (74.5)	<0.001
**Understanding of HIV services (full score 8)/Median score (IQR)**	4 (2,5)	7 (6,7)	8 (6,8)	<0.001*
**% median composite score (%IQR)**	50 (25, 62.5)	87.5 (75, 87.5)	100 (75, 100)	
State the name of your physician, clinic, and next appointment date	35 (21.7)	103 (64.0)	125 (77.6)	<0.001
Know what to do if your ARV run out before the next appointment (knowing how to make an appointment)	93 (57.8)	151 (93.8)	156 (96.9)	<0.001
State symptoms requiring a doctor's visit	84 (52.2)	144 (89.4)	152 (94.4)	<0.001
State your health insurance coverage	107 (66.5)	150 (93.2)	157 (97.5)	<0.001
Describe the process of requesting a medical referral	69 (42.9)	126 (78.3)	140 (87.0)	<0.001
Describe the process treatment services are obtained and given	140 (87.0)	157 (97.5)	157 (97.5)	<0.001
State the reason(s) for transition to adult clinic	69 (42.9)	137 (85.1)	155 (96.3)	<0.001
State at least two organizations where social support and emergency help are provided	20 (12.4)	53 (32.9)	97 (60.2)	<0.001
**Reproductive Health and Family Planning (full score 3)/Median (IQR)**	2 (1, 2.5)	3 (3, 3)	3 (3, 3)	<0.001*
**% median composite score (%IQR)**	66.7 (33.3, 83.3)	100 (100, 100)	100 (100, 100)	
State at least two birth control methods	126 (78.3)	158 (98.1)	158 (98.1)	<0.001
Describe methods to prevent transmission of sexually transmitted infections and HIV	129 (80.1)	158 (98.1)	158 (98.1)	<0.001
Describe how to put on a condom correctly	43 (26.7)	140 (87.0)	153 (95.0)	<0.001
**Career and Education (full score 1)/Median (IQR)**	1 (0, 1)	1 (1, 1)	1 (1, 1)	<0.001*
**% median composite score (%IQR)**	100 (0, 100)	100 (100, 100)	100 (100, 100)	
Know how to get information about education and careers	120 (74.5)	152 (94.4)	157 (97.5)	<0.001
**Total score (22)/Median (IQR)^a^**	10 (7,12)	18 (15,20)	21 (18.5, 22)	<0.001*
**% median composite score (%IQR)**	45 (31.8, 54.5)	81.8 (68.1, 90.9)	95.5 (84.1, 100)	

IQR interquartile range. Statistical analyses using Cochran's Q‐Test. *Statistical analyses using Friedman Test. ^a^The significance cut point (α = 0.05) using the Bonferroni correction for multiple testing is <0.0028.

The baseline score for youth who participated in the HT1 programme (median 11 [IQR 10–13]) was higher than score of youth who did not participate (median 9 [IQR 7–11],*P* < 0.01) and the baseline score of youth participating at Siriraj Hospital (median 10 [IQR 9–13]) was higher than youth at QSNICH (9 [IQR 6–11], *P* < 0.01). After participation in the HT2 programme, the median knowledge score for ARV management (e.g. current ARV, adherence, side effects) increased from 3 (75%) of 4 at baseline and 12 months to 4 (100%) of 4 at 18 months (*P* <0.001). Median score for knowledge about HIV plasma viral load (VL) and CD4 count results increased from 0 (0%) of 6 at baseline to 4 (67%) of 6 at 12 months to 6 (100%) of 6 at 18 months (*P* <0.001). The score for understanding how and when to access HIV care increased from 4 (50%) of 8 at baseline to 7 (88%) of 8 at 12 months to 8 (100%) of 8 at 18 months (*P* <0.001). Knowledge of reproductive health and family planning increased from 2 (67%) of 3 at baseline to 3 (100%) of 3 at 12 and 18 months (*P* <0.001). At baseline and upon completing the intervention, most youth were able to describe where to obtain information about educational opportunities and careers. Eighteen months after the intervention, 40% of youth were still not able to state the names of organizations providing social support or emergency services, 25% did not know their VL level, 22% did not know the target value for VL, 22% were unable to state the name of their physician, clinic, and next appointment date, and 20% were unable to state the name of their current ARV.

In multivariable analysis, an overall knowledge score at 18 months >95% was associated with an education level higher than high school (adjusted odds ratio [aOR]: 2.15; 95% confidence interval [CI], 1.03–4.48) and receipt care at QSNICH (aOR: 2.43; 95% CI, 1.18–4.98). Youth whose parents (i.e. mother and father) had died were less likely to have score ≥95% at 18 months compared to youth whose parents were alive (aOR: 0.22; 95% CI, 0.07–0.67) (supplemental table 5).

As of December 2016, 134 (83%) of the 161 youth have returned for follow‐up, one year after completing the programme. Of these, 24 (18%) youth (median age of 17 years) had transferred to an adult HIV clinic. All youths who transitioned to adult HIV care services reported high satisfaction of the HT2 programme and reported that the knowledge gained from the programme was helpful during the transition process. All of youths were still on ART and in follow‐up at the adult clinics.

### Discussion

We developed the Happy Teen programme as a holistic outpatient clinic‐based approach to support HIV‐infected youth as they transition from paediatric to adult care services. HIV‐infected youth participating in the HT2 programme for 18 months showed significant improvements in knowledge of ARV management, the meaning of CD4 count and VL, and their understanding of HIV services, all factors important [1,14] for a successful transition to adult HIV care services [1,4,15]. We found that youth had low overall knowledge scores at baseline despite long‐term care in HIV paediatric clinics at tertiary care hospitals [12] and recognize the importance of implementing activities to provide youth knowledge required for self‐care and a successful transition to adult HIV services [1,16,17]. The programme received high satisfaction scores from perinatally HIV‐infected youth, the participation rate in the individual sessions was high, and the model was feasible to implement and conduct during routine clinic visits.

As perinatally HIV‐infected youth age and their time on ART increases [14], some experience changes in or complications from ART [1]; emotional and behavioural problems are more common in this adolescent group than among the general population [18]. As with other teenagers, youth with perinatal HIV infection engage in sexual intercourse, but unless they use condoms correctly, they can transmit HIV to their sexual partners [19,20]. Therefore, youth may need to consider disclosing HIV status to their sexual partners. The Happy Teen programme covers four strategic areas (i.e. health knowledge, coping skills, sexual risk reduction, and life goals) with both group and individual activity sessions. Group sessions allow youth, adult caretakers, and paediatric healthcare providers to participate in the activities. The aim is to promote youth self‐confidence, improve the communication skills of youth and their caretakers, and to allow youth to share experiences and receive support from their peers and caretakers for issues related to sexual reproductive health, career plans, and HIV care [10]. The programme engaged adult providers to participate in the one‐day workshop and half‐day meetings so that adult providers and youth could work together to coordinate the transition from one provider to another [1,2,21,22]. In Thailand, many youth live with their parents or relatives and are under the direct supervision of adult caretakers. In our study, more than 90% of youth were living with parents or relatives. During the HT1 programme some youth reported communication problems with their caretakers regarding their HIV care. Thus, we felt it was important to engage caretakers in the HT2 programme to help them understand factors important in the transition from paediatric to adult HIV care services and to provide an opportunity to improve communication in the family [3,6,23]. We found that youth whose parents had died had lower knowledge scores at 18 months compared to those whose parents were alive. This is in line with research that has shown that parental engagement has a positive impact on student achievement and literacy [24,25], suggesting that parental involvement and family‐based interventions play an important role in achieving optimal continuum of care for transitioning of youth living with HIV [3,21,26].

Study participants reported that they enjoyed caretaker involvement in activities which sought to improve communication within families. However, the findings revealed that over‐engagement by caretakers had negative consequences on achieving the development of good self‐care habits among youth participants [27]. Additionally, we found that the presence of parents at group sessions, particularly sessions related to sexual reproductive health, hindered youth participation and engagement in the programme due to increased self‐consciousness and shyness.

Only 29% of youth attended all the sessions and completed the entire programme. Almost half of the youth were not able to attend the group activities including the one‐day workshop that was organized as an extra‐visit, but more than 90% were able to attend the individual sessions that were integrated into the routine appointment schedule. The individual sessions which lasted 30–60 min provided an opportunity to assess youths’ knowledge gaps in the four strategic areas and offer youth‐focused interventions to promote ART adherence, risk reduction, and retention in care during the transition period [28]. Although clinical outcomes were better at Siriraj and median baseline knowledge score was higher at Siriraj Hospital (data not shown), the knowledge score at 18 months was higher at QSNICH. A higher proportion of participants at QSNICH completed the entire programme and attended more sessions, including the half‐day workshops, than participants at Siriraj Hospital. This may be because QSNICH's HIV clinic operates in the afternoon, allowing youth to come to the clinic in the morning for blood collection, participate in the half‐day meeting, and then see the doctor in the afternoon. The HIV clinic at Siriraj Hospital operates in the morning and youth may have decided not to stay for the half‐day meeting in the afternoon.

We found that youth retained knowledge when given an opportunity to practice (e.g. youth were given an opportunity to call the emergency line number during the individual session). Youth who achieved a higher level of education had a higher knowledge score than those with less education, suggesting that factors associated with academic success also contribute to health knowledge [29].

There is no defined age when youth transfer to adult care at the two hospitals in this study. Transition decisions are based on youth readiness, their health status (e.g. if a person requires hospital admission and is >15 years old, they are admitted to the adult service), and changes in healthcare insurance scheme (e.g. youth who are employed may be required to change registration hospital) [1,13]. In Thailand, some hospitals transfer youth living with HIV to adult care at the age of 15 years. The Thai guidelines for transitioning youth from paediatric to adult HIV care services recommend that preparations begin at least one year before the planned transition to help adolescents progress towards self‐management [13] and to encourage communications among youth, families/caretakers, and their providers for transition plan [8,13,22,27].

This study had several limitations. Almost all youth participating in the HT2 programme were perinatally HIV infected and we do not know if the programme would have similar results among youth with behaviourally acquired infections. We only measured outcomes in participants who completed the 3 questionnaires and may have selected a particularly motivated population of youth. To correct this bias, we performed a sensitivity analysis using a pair‐wise McNemar test of 175 youth who completed the baseline and 12 month questionnaire and the 161 youth who completed the 12 month and 18 month questionnaires and the results still showed significant improvement in knowledge scores. About a third of the youth in the HT2 programme participated in the HT1 programme which may have primed them to achieve better knowledge scores at baseline. Nonetheless, youth in the HT2 programme showed significant increases in knowledge scores from baseline to month 18. We did not have a control group in this assessment, but we found that youth who participated in more sessions (i.e. QSNICH's participants) had better scores at 18 months. Because we administered the same questionnaire to participants three times, it is not clear if participants overall knowledge about HIV and self‐care improved or if they memorized the answers to the questions. Long‐term follow‐up of youth will be necessary to assess their transition to adult medical services and determine if there is a relationship between the knowledge scores and a successful transition. We will continue to follow the youth who participated in the HT2 programme to assess behaviours and medical outcomes following the transition from paediatric to adult medical services.

### Conclusions

The Happy Teen programme for HIV‐infected youth has been successfully implemented in two tertiary care hospitals in Bangkok. We found that youth aged 14–22 years old were satisfied with the programme and were able to demonstrate improved knowledge of HIV care and care services after participating in the program. The programme was feasible to implement during routine clinical services. The longer‐term impact on behavioural outcomes and the successful transition of youth to adult HIV care services needs further study.

## Competing interests

The authors declared no competing interests.

## Authors’ contributions

RL participated in study design, project implementation, statistical analysis, interpretation of data, and drafting and revision of the manuscript. KC participated in study design, project implementation, data collection, interpretation of data, and revision of the manuscript. VB, YN, BM, WP, JT, WN, PL, KL, PS participated in study design, project implementation, and data collection. MM participated in study design, statistical analysis, interpretation of data, and revision of the manuscript. VT performed data analysis. All authors reviewed and approved the final version of the manuscript.

## Acknowledgements

We acknowledge the Happy Teen programme working group at Siriraj Hospital: Yuitiang Durier and Sirijit Kanakool, at QSNICH: Dr. Pakpen Sirikutt, Nisachol Ounjit, Sujinda Yadech, and at TUC: Chanidapa Yuvasevee, Thananda Naiwatanakul, Dr. Thierry Roels, and the staff of the paediatric department, Siriraj hospital and QSNICH for their contribution to this project. We express our gratitude to all youth and caretakers participating in this project. We sincerely thank Philip Mock for his support of the data analysis.

To access the supplementary material to this article please see http://doi.org/10.7448/IAS.20.4.21500 under Article Tools online.

## Supporting information

Supplemental Table 5
