# Supplementary material for: The Happy Teen programme: a holistic outpatient clinic‐based approach to prepare HIV‐infected youth for the transition from paediatric to adult medical care services in Thailand
Source: J Int AIDS Soc. 2017 May 16;20(Suppl 3):21500. doi: 10.7448/IAS.20.4.21500 (PMC5704900; doi:10.7448/IAS.20.4.21500)
Supplement: Supplementary file 1 — Supplemental Table 5 [file JIA2-20-21500-s001.docx]

**Supplemental Table 5: Factors associated with a knowledge score >95 percent among participants (n=161) in the Happy Teen 2 Program at 18 months, Thailand 2015-2016.**

| **Factor** | **Score >95 %** | | **Bivariable analysis** | | | | **Multivariable analysis** | | | |
| --- | --- | --- | --- | --- | --- | --- | --- | --- | --- | --- |
|  | **n** | **%** | **Odds ratio** | **95% CI** | | **p-value^a^** | **Odds ratio** | **95% CI** | | **p-value^a^** |
|  |  |  |  | **Lower** | **Upper** |  |  | **Lower** | **Upper** |  |
| 1. Hospital |  |  |  |  |  |  |  |  |  |  |
| - Siriraj | 20 | 33.3 | 1 |  |  |  | 1 |  |  |  |
| - QSNICH | 40 | 66.7 | 2.12 | 1.09 | 4.12 | 0.026 | 2.43 | 1.18 | 4.98 | 0.017 |
| 2. Age |  |  |  |  |  |  |  |  |  |  |
| - <17 yrs | 25 | 41.7 | 1 |  |  |  |  |  |  |  |
| - ≥17 yrs | 35 | 58.3 | 1.08 | 0.57 | 2.06 | 0.814 |  |  |  |  |
| 3. Gender |  |  |  |  |  |  |  |  |  |  |
| - Female | 22 | 36.7 | 1 |  |  |  | 1 |  |  |  |
| - Male | 38 | 63.3 | 1.83 | 0.95 | 3.53 | 0.069 | 1.96 | 0.96 | 4.21 | 0.067 |
| 4. Occupation |  |  |  |  |  |  |  |  |  |  |
| - Unemployment | 1 | 1.7 | 1 |  |  |  | 1 |  |  |  |
| - Employee | 3 | 5.0 | 3.54 | 0.32 | 39.14 | 0.302 | 4.26 | 0.35 | 51.6 | 0.255 |
| - Student, Non-formal education student d | 56 | 93.3 | 9.46 | 1.20 | 74.39 | 0.033 | 5.38 | 0.64 | 45.58 | 0.122 |
| 5. Highest Education level |  |  |  |  |  |  |  |  |  |  |
| - None, Primary, Junior high school, Other | 20 | 33.3 | 1  1. |  |  |  | 1 |  |  |  |
| - Bachelor degree, High school | 40 | 66.7 | 2.30 | 1.18 | 4.46 | 0.014 | 2.15 | 1.03 | 4.48 | 0.041 |
| 6. Literacy |  |  |  |  |  |  |  |  |  |  |
| - Fair, Illiterate | 5 | 8.3 | 1 |  |  |  |  |  |  |  |
| - Very good | 55 | 91.7 | 2.55 | 0.90 | 7.23 | 0.079 |  |  |  |  |
| 7. Main Caretaker(s) |  |  |  |  |  |  |  |  |  |  |
| - Others (orphanage, self-care) | 3 | 5.0 | 1 |  |  |  |  |  |  |  |
| - Relatives | 27 | 45.0 | 2.11 | 0.54 | 8.22 | 0.284 |  |  |  |  |
| - Parents | 30 | 50.0 | 2.56 | 0.66 | 9.96 | 0.176 |  |  |  |  |
| 8. Parents Status |  |  |  |  |  |  |  |  |  |  |
| - Both alive | 18 | 30.0 | 1 |  |  |  | 1 |  |  |  |
| - Both dead | 8 | 13.3 | 0.30 | 0.11 | 0.81 | 0.018 | 0.22 | 0.07 | 0.67 | 0.008 |
| - One parent dead, Unknown | 34 | 56.7 | 0.76 | 0.35 | 1.63 | 0.476 | 0.60 | 0.26 | 1.39 | 0.233 |
| 9. Monthly household income (per month) |  |  |  |  |  |  |  |  |  |  |
| - <20,000 baht | 47 | 78.3 | 1 |  |  |  |  |  |  |  |
| - >20,000 baht | 13 | 21.7 | 0.89 | 0.41 | 1.91 | 0.760 |  |  |  |  |
| 10. Period from HIV disclosure to enrollment (months) | - | - | 1.01 | 1 | 1.02 | 0.115 |  |  |  |  |
| 11. Participated in Happy Teen 1 Program |  |  |  |  |  |  |  |  |  |  |
| - No | 41 | 68.3 | 1 |  |  |  |  |  |  |  |
| - Yes | 19 | 31.7 | 0.91 | 0.46 | 1.81 | 0.794 |  |  |  |  |
| 12. Time in HIV care (years): *median=13* |  |  |  |  |  |  |  |  |  |  |
| - < median | 24 | 40.0 | 1 |  |  |  |  |  |  |  |
| - > median | 36 | 60.0 | 1.47 | 0.77 | 2.81 | 0.243 |  |  |  |  |
| 13. Period of ARV use (years): *median=13* |  |  |  |  |  |  |  |  |  |  |
| - < median | 26 | 43.3 | 1 |  |  |  |  |  |  |  |
| - > median | 34 | 56.7 | 1.44 | 0.76 | 2.75 | 0.263 |  |  |  |  |
| 14. Plasma HIV RNA concentration |  |  |  |  |  |  |  |  |  |  |
| - VL <40 copies/ml | 48 | 80.0 | 1 |  |  |  |  |  |  |  |
| - VL >= 40 copies/ml | 12 | 20.0 | 0.80 | 0.37 | 1.75 | 0.546 |  |  |  |  |
| 15. Participated in all sessions |  |  |  |  |  |  |  |  |  |  |
| - Not all sessions | 36 | 60.0 | 1 |  |  |  |  |  |  |  |
| - All sessions | 24 | 40.0 | 1.44 | 0.74 | 2.8 | 0.285 |  |  |  |  |

***Note****: CI = confidence interval*

*^a^ p-values for logistic regression*

*All factors adjusted for hospital, gender, occupation, highest education and parent status.*
